# Understanding the gender gap in advanced practice nursing: A qualitative study

**DOI:** 10.1111/jonm.13886

**Published:** 2022-11-24

**Authors:** Brigitte Fong Yeong Woo, Yong Shian Goh, Wentao Zhou

**Affiliations:** ^1^ Alice Lee Centre for Nursing Studies, Yong Loo Lin School of Medicine National University of Singapore Singapore; ^2^ National Neuroscience Institute Singapore

**Keywords:** nurse practitioners, nursing image, recruitment, workforce

## Abstract

**Aims:**

We aim to explore the perceptions of registered nurses undergoing the advanced practice nurse preparatory programme and of advanced practice nurses towards the recruitment of men into the advanced practice nursing workforce.

**Background:**

Given the need to expand and diversify the advanced practice nursing workforce, it is important to recognize the potential implications of gender disparity. It is critical to understand why few males are recruited into the advanced practice nursing preparatory programme and to determine whether gender‐related bias is present in the professional development of nurses.

**Method:**

We use a descriptive qualitative design. Semi‐structured interviews were conducted via Zoom (April to August 2021).

**Results:**

Three themes were generated: ‘The odds are eventually in the favour of men’, ‘The APN career‐track is unpopular among men’, and ‘Balancing the gender gap in the APN workforce’. All themes are supported by three subthemes.

**Conclusions:**

Although males in nursing enjoy opportunistic advantages in entering the advanced practice nursing workforce, they are not interested in the role and not staying long enough in the nursing profession to become advanced practice nurses.

**Implications for Nursing Management:**

Nurse managers should be more cognizant of the different social stereotypes faced by males and females in nursing. With such awareness, they can be more supportive and less biased in career counselling and job appraisals.

## 1 INTRODUCTION

Gender disparity has long been noted in nursing, a predominantly female‐dominated profession (Barrett‐Landau & Henle, 2014). Across the world, males comprise only 11% of the nursing workforce (World Health Organization, 2020). This is unsettling because an accurate representation of the general population's demographic composition is integral to any profession. Furthermore, such disparity is of concern especially in view of the global shortage of nursing staff: The World Health Organization (2021) has forecast a global shortfall of 9 million nurses to meet population health needs by 2030.

In Singapore, gender disparity has persisted. Although the number of male nurses has risen with time, the pace is not encouraging: their proportion among registered nurses (RNs) has only increased from 8.2% in 2008 to 11.6% in 2020 (Singapore Nursing Board, 2021). The phenomenon is also true among Singapore's advanced practice nurses (APNs), who are nursing leaders spearheading patient care with demonstrable clinical contributions (Laurant et al., 2018; Smits et al., 2020; Woo et al., 2017). The proportion of males among APNs remains disproportionately low: they currently account for less than 5% of the APN workforce (Singapore Nursing Board, 2021).

### 1.1 Men in nursing

Nursing being a female‐dominated profession has precipitated men in nursing to experience gender role conflicts, stereotypes, and challenges in educational and clinical settings (Younas et al., 2019; Zeb et al., 2020). Men in nursing started encountering such negative experiences while they were students. One common assumption held by many is that men who end up pursuing nursing are only doing so because they are not bright enough to become doctors (Brody et al., 2017). Often, they are also questioned about their sexual orientation (Brody et al., 2017). As a result, male nursing students reportedly have concerns and uncertainties about becoming a nurse. They fear poor acceptance of male nurses among healthcare colleagues and patients would negatively impact their career prospects (Younas et al., 2019; Zeb et al., 2020).

Stereotypes about masculinity have influenced nursing care assignments in clinical practice. Men in nursing are expected to work in fast‐paced clinical environments, such as intensive care units or emergency departments, where there is more heavy lifting (Cheng et al., 2018). Men are also thought to be better suited for psychiatry settings where their physical strength would be helpful in managing patients who are at risk to themselves and others (Jordal & Heggen, 2015). In some contexts, men are outrightly precluded from caring for female patients (Zeb et al., 2020). These role expectations can marginalize and cause a strain among men in nursing (Smith et al., 2020).

To avoid gender role conflicts and discrimination, some men become nurse educators. Although these men received more respect and appreciation as educators, they still feel compelled to constantly prove their worth and improve themselves. These feelings stemmed from prejudices engrained in the nursing culture against male nurses (Zeb et al., 2020). Over time, these issues men face in nursing could culminate and result in feelings of isolation, stress, and burnout (Carnevale & Priode, 2018). These could negatively impact retaining men in nursing (Rajacich et al., 2013).

### 1.2 Recruitment of APNs in Singapore

In Singapore, APNs are Masters‐prepared nurses with a professional licence to practice beyond an RN's fundamental scope of practice. The role of an APN in the context of Singapore represents a hybrid between that of a nurse practitioner and a clinical nurse specialist (Woo et al., 2019). Additionally, some APNs have collaborative prescribing authority (Ministry of Health, 2018).

Currently, the Singapore Nursing Board oversees the APN practice and licensure, the prerequisite for which is a formal preparatory training. This comprises an 18‐month Master of Nursing programme at the National University of Singapore and a 12‐month supervised internship under a provisional licence (Singapore Nursing Board, 2020). Upstream enrolment of RNs into the Master of Nursing programme requires endorsement and recommendation from their nursing leaders (besides minimum academic requirements and clinical experiences) (National University of Singapore, n.d.). An aspect unique to Singapore is that such matriculated RNs will enter an employment bond with their healthcare institutions, under which they are thus expected to develop their careers as APNs. Accordingly, nursing leaders are crucially involved in selecting RNs for APN practice (Woo et al., 2020).

A robust nursing workforce is critical to attaining universal health. Thus, there has been an ongoing interest to recruit more APNs to strengthen the APN workforce, as demonstrated by the aim of Singapore's Ministry of Health to double the number of APNs by 2030 (Begum, 2021). Accordingly, expanding the APN workforce entails addressing the gender disparity, especially given the evolving healthcare and sociocultural landscape (Thompson et al., 2020). Moreover, diversifying the APN workforce translates into the practical utility of tapping into the male population for their expertise. This builds a more gender‐inclusive environment for males and ultimately improves patient care and experiences (Smith et al., 2020).

Given the need to expand and diversify the APN workforce, it is important to recognize the potential implications of gender disparity. Therefore, it is critical not only to understand why few males are recruited into the APN preparatory programme but also to determine whether gender‐related bias is present in the professional development of RNs. Furthermore, it may be propitious to understand if men are staying long enough in nursing to become APNs. Such findings are envisioned to provide practical insights into potential solutions to address gender disparity.

### 1.3 Aims

Accordingly, this inquiry aimed to explore the perceptions of RNs undergoing the APN preparatory programme and of APNs towards:

- the shortage of men in the APN workforce;
- the selection and recruitment of men into the APN preparatory programme; and
- the influence of having more men in the APN workforce on the professional image of nursing in Singapore.

## 2 METHODS

### 2.1 Study design

A descriptive qualitative design (Polit & Beck, 2012) was adopted. Participant recruitment, data collection, and data analysis were iteratively performed to explore diversity from the nurses' perspectives. Additionally, this study adhered to the 32‐item Consolidated criteria for Reporting Qualitative research (Tong et al., 2007) (Appendix S1).

### 2.2 Setting and sample

Singapore, a city‐state with a total population of 5.45 million people and a population density of 7485 people per square kilometre, was the study setting (Singapore Department of Statistics, 2022). There are 35,948 RNs (32,621 in active practice) and 330 APNs (327 in active practice) in Singapore (Singapore Department of Statistics, 2022).

The participants were purposively sampled. Included in this study were consenting male and female registered APNs, APN interns, and Master of Nursing students aged 21 years and above. Within the context of this study, the term ‘APNs’ refers to nurses who have completed all aspects of the formal APN preparatory training. ‘APN interns’ refers to Master of Nursing graduates in the midst of their 12‐month supervised internship under a provisional licence. Finally, ‘Master of Nursing students’ refers to RNs receiving formal APN education at the university. Participants must have access to a personal computer or mobile device with microphone and camera functions. APNs, APN interns, or Master of Nursing students who refused to have their interviews audio‐recorded or their web cameras turned on during the videoconference were excluded.

Electronic invitation letters with information about the study were disseminated via email to all the National University of Singapore Master of Nursing alumni by the programme administrator. In addition, interested alumni were requested to contact the study team via email. The study team would then respond and arrange a mutually convenient time to go through the consent process and conduct the interview. Snowballing sampling was also adopted. The study team also received referrals from the study participants.

### 2.3 Data collection

Each consenting participant then took part in a single session, individual, semi‐structured interview through videoconferencing (Zoom) between April and August 2021 with one of the authors (B. W., Female, PhD‐prepared). During the interviews, web cameras and microphones were set up so that the researcher and participants could see and hear each other (including the participants' non‐verbal cues). The researcher had no prior relationships with the participants.

With a flexible interview guide, the researcher explored the participants' perspectives towards males in the APN workforce. Examples of the questions were: ‘Why is there an under‐representation of males in the APN workforce?’ and ‘Do you think nursing leaders take into consideration the gender of the RN when nominating him or her for the MN programme?’. The interviews were audio‐recorded and transcribed in verbatim, ranging from 48 to 64 min (median = 54 min).

### 2.4 Data analysis

Data analysis and data collection took place in parallel and complemented each other. During data analysis, no new information and codes were generated between the 15th and 16th interviews. It was then the study team agreed that data saturation was achieved (Green & Thorogood, 2004). Accordingly, recruitment concluded at the 16th interview. Following this, thematic analysis, guided by Braun and Clarke (2006), was undertaken inductively, with the themes and subthemes being extrapolated from the data rather than from existing conceptual frameworks or theories (Fereday & Muir‐Cochrane, 2006).

When generating codes across the data corpus, some techniques from the grounded theory were used (Charmaz, 2014). The techniques included line‐by‐line coding of the interviews transcribed in verbatim, discussions among study team researchers about emergent codes and deviant cases, and adopting a constant comparative method (Strauss & Corbin, 1990). The researchers read the interview transcripts and listened to the audio‐recordings repeatedly to gain familiarity with the data. Next, codes were refined and arranged into potential themes and subthemes using a semantic approach. The themes and subthemes were assessed if they cohere meaningfully with the codes and data corpus. Subsequently, the themes and subthemes were refined and defined (Braun & Clarke, 2006). The constant comparative method was undertaken in coding, developing, reviewing, and defining themes. The relationships between and within each theme were compared (Strauss & Corbin, 1990). Discussions continued until all the researchers (B. W., G. Y. S., and Z. W.) agreed on the final themes and subthemes (Braun & Clarke, 2006).

### 2.5 Rigour

To ensure the rigour of our study, criteria for authenticity and trustworthiness were upheld (Guba & Lincoln, 2018). Authenticity was ensured by exercising fairness, demonstrated by assigning equal consideration and weight to every participant's insights (Guba & Lincoln, 2018). Trustworthiness involves credibility, dependability, confirmability, and transferability. Each interview lasted more than 30 min to ensure credibility, allowing prolonged engagement with the participant. Additionally, to enable triangulation, the data analysis was undertaken by three researchers (B. W., G. Y. S., and Z. W.).

Furthermore, member‐checking followed the interviews, involving four consenting participants with contrary perspectives: They were first presented with the preliminary themes, subthemes and participants' quotes. Then, they were requested to examine the representativeness of their views (Birt et al., 2016). To ensure dependability and confirmability, the interview transcripts were checked by researchers not involved in transcription. Throughout the data analysis, an audit trail was kept alongside field notes and reflexive annotations to record the researchers' tendencies and intents. Lastly, to permit readers to determine the transferability of this study, the participants' socio‐demographic characteristics, verbatim quotes, and relevant contexts were provided for their appraisal.

## 3 FINDINGS

### 3.1 Demographic profile

Of the 16 participants recruited (eight males and eight females), the majority were Chinese (*n* = 13) and were married (*n* = 12). Their ages ranged from 29 to 43 years (median = 36 years), and their nursing experiences ranged from 6 to 20 years (median = 11 years) (Table 1). Six were Master of Nursing students, three were in the midst of their APN internships, and seven were APNs. All but two of them were working with adult patients.

**TABLE 1:** Participant characteristics (*n* = 16)

Demographics	*n*
***Age (years)***	
Median (range)	36 (29–43)
***Gender***	
Female	8
Male	8
***Race***	
Chinese	13
Indian	2
Malay	1
***Marital status***	
Married	12
Single	4
***Nursing work experience (years)***	
Median (range)	11 (6–20)
***APN career stage***	
MN student	6
APN intern	3
APN	7
***Highest level of education***	
Master's degree	10
Bachelor's degree	5
Post‐graduate diploma	1
***Current job title***	
Senior staff nurse	8
Assistant nurse clinician	1
Nurse clinician	3
Senior nurse clinician	3
Assistant director of nursing	1
***Work setting***	
Adult inpatient	6
Adult acute care (ICU or ED)	4
Paediatrics (inpatient)	2
Mental health (inpatient)	2
Mental health (inpatient, outpatient and community)	1
Adult inpatient and outpatient	1

Abbreviations: MN, Master of Nursing; APN, Advanced Practice Nurse; ICU, intensive care unit; ED, emergency department.

### 3.2 Thematic findings

Three themes were generated, each of which was, in turn, supported by three subthemes that further illustrated the participants' perspectives towards the selection and recruitment of males into the APN workforce (Table 2).

**TABLE 2:** Themes and subthemes

Themes	Subthemes	Quotes
The odds are eventually in the favour of men	Objectivity in the selection process	‘I do not think nursing leaders will take into account the gender. I think they look more into GPAs, so like they will consider your education background, and you are your contributions to the organisation, and how well have you performed. You have to score A for 3 consecutive years for your work appraisal and your GPA must be 3.2 and above. *[*sic*]*’ (P16, female, MN student) ‘I think it depends more on their academic results, their calibre and suitability for the APN role rather than gender issue *[*sic*]*.’ (P13, male, APN)
	Differing gender‐specific role expectations in the family	‘That [the need to defer plans to get pregnant] caused one of the potential candidates actually to turn down the offer [to enter the MN programme] … ambiguity to when she can start family planning caused her to withdraw from the programme and prioritise family planning over, umm, the APN programme. *[*sic*]*’ (P1, male, student) ‘… when they [women] have to deliver [the baby], of course, they will be unable to carry on with their studies. So, they either have to postpone it, or some of them, if they cannot cope with their family, their kids, I'm not sure whether they need to withdraw from the programme. But this is not part of my considerations. *[*sic*]*’ (P3, male, student)
	The inevitable ‘glass escalator’	‘I feel like even though male nurses are the minority, there's certain advantage. So, I've seen male nurses if you [men] are good right, you are recognised more easily than your female counterparts because they [men] are so scarce, right? So, if one stood out, that person have more opportunities compared to his peers who are female, because he just stands out more easily. He′s male and he can just outshine … he just managed to, in a way, get the upper hand. *[*sic*]*’ (P4, female, intern) ‘The career progression [of men] is smoother, as long as they do not cause a kerfuffle at work. *[*sic*]*’ (P1, male, student).
The APN career‐track is unpopular among men	Gender‐stereotyped attributes influencing career choice	‘… stereotype that males have better management style because they are more task‐oriented and less emotional in their decision making makes male nurses better nurse managers. *[*sic*]*’ (P1, male, student) ‘I somehow think it is better for males to become managers. Males are less reactive in a lot of circumstance, so always give people the vibe that they are a bit more cool‐headed. So, I think that is a good leadership quality … more cool‐headed, less reactive. So, I feel that males usually in the female environment they tend to have a better head start. I feel male have a better head start of the management or leadership pathway. *[*sic*]*’ (P5, female, student)
	Sacrifices in renumeration and career progression	‘… by the 4th year, you are just starting to craft your role as an APN … compared to the regular RNs who stay in clinical and continue to provide their service [to the organisation]. Definitely, there will be more opportunities for them [regular RNs] to get promoted compared those who decided to go for the MN programme. *[*sic*]*’ (P9, female, APN) ‘After you graduate as an APN, your increment around 400 to 450, right? But you spend almost three years to struggle with this [MN] program. But if they [RNs] stay in the clinical area, if they promote as an NC [nurse clinician] or ANC [assistant nurse clinician], they can get seven hundred dollars … one of my NC currently in my ward, he does not want to go to be an APN. He went to study a masters of management in one of those private universities. He thinks, ‘why do I need to struggle? My pay going to increase more than your salary.’ *[*sic*]*’ (P14, male, intern)
	Gender‐based stigmatisation of nursing	‘I have brothers. If I were to ask my brothers to consider nursing as a profession, they would say, ‘oh, it's a girl's job.’ So I mean, as much as I do not want people to think that way, but I cannot, you know? It's something is in‐built in them that nursing is very feminine, cannot have men in doing the job. Then any men who is in nursing are thought to be feminine and not masculine. *[*sic*]*’(P15, female, intern) ‘… in Asian countries, maybe China and Taiwan, I think there is still this mindset that being a nurse is still a female‐dominated work. *[*sic*]*’ (P3, male, student)
Balancing the gender gap in the APN workforce	Male APNs are a ‘good‐to‐have’ and not a ‘must‐have’	‘There is not really anything different what male and female APNs are doing. That is quite awesome. *[*sic*]*’ (P12, male, APN) ‘I think there are some ways where guys think a little bit differently from females … I think we appreciate the way how different people think … a team cannot be of the same character or same personality or the same way of thinking because you will not have change, you will improve. *[*sic*]*’ (P7, female, APN)
	Good role models and better articulation of the role	‘… seeing how our APN seniors work is an inspiration to us. We see how they are equipped with masters and being APN, they do more for our patients. They have little bit more autonomy at work. APNs can make certain decisions without needing to ask permissions from doctors … more autonomy in terms of providing care. *[*sic*]*’ (P13, male, APN) ‘There are still a lot of questions revolving around what is the service that they [APNs] provide … they have to differentiate themselves from the nurse clinicians … it requires quite a lot of pioneering kind of work to tell your colleagues what your role as an APN is and what you are working for. *[*sic*]*’ (P7, female, APN)
	‘De‐gendering’ the gendered stigmatisation of nursing	‘[having more men] dispels the social stigma of nursing being an effeminate job. *[*sic*]*’ (P5, female, student) ‘… we do need a male representative to show that our job, there is nothing soft about it. There's nothing feminine about it. It's actually very tough. So having a guy in the APN profession or to represent nursing or to represent APN can actually show people, ‘oh nursing is not an easy job. It's can be a masculine job.’ It actually takes lots of guts and lots of IQ [intelligence quotient] and EQ [emotional quotient]. *[*sic*]* (P15FI)

#### 3.2.1 Theme 1: The odds are eventually in the favour of men

The participants shared that there was no gender preference in the selection and recruitment of APNs into the workforce. However, they opined that males might enjoy opportunistic advantages over females in the selection for the APN candidature.

##### Subtheme: Objectivity in the selection process

In general, there is a stipulated set of objective criteria for selecting nurses for the formal APN preparatory education. According to the participants, any nurse might express their interest in the APN track to their direct reporting officers (usually the nurse managers or nurse clinicians of their wards or clinics). Following this, the managers or clinicians would submit a nomination for the interested nurse. Some participants shared that their institutions made application forms available on the intranet for interested nurses. Others added that potential candidates in their institutions were to be assessed for clinical acumen and critical thinking through short assessments known as Multiple Mini Interviews:

… the selection process is objective because we use the MMI [multiple mini interviews]. So, it is an objective way to assess the clinical ability of a nurse. So, it is a voluntary kind of sign up like whoever is considering to become an APN, they just need to sign up. Basically, everybody has equal chance … *[*sic*]* (P8, Female, APN)

Additionally, the participants remarked upon the need for interviews with their nursing leaders and Human Resource personnel. During these interviews, candidates would be evaluated for not only their understanding of and interest in the APN role but also their suitability for it. The need for APN‐led services in a given specialty was usually examined as well:

… although you are may be interested in a certain area but that area of expertise may not require an APN … they [nursing leaders] said, ‘These are the few disciplines that we are currently accepting APNs. Do you feel you are interested in any of these?’ *[*sic*]* (P2, Male, student)

In sum, the selection and assessment of the candidates by the nursing leaders and administrators were reported by the participants to be independent of the candidates' gender.

##### Subtheme: Differing gender‐specific role expectations in the family

According to the participants, during the selection interviews, the candidates were frequently asked questions related to their families and the need for help at home while they embarked on the APN preparatory training:

… am I married, single? Do I have children? Who is looking after the children? Am I or will I be occupied with family? *[*sic*]* (P6, Male, Student)

they were very concerned [that I have a toddler]. One of the interview questions I got asked, I think twice, was whether or not I am able to cope with being a mother and a full‐time student. That's the question they [nursing leaders] asked during the interview. *[*sic*]* (P15, Female, Intern)

Given its intensive nature, the APN preparatory training was known to demand full commitment from the candidates and might even require time away from their families. Hence, family support was deemed an important contributor to the successful completion of the programme. In this regard, married female candidates were strongly encouraged to delay plans of pregnancy because it might disrupt the training. Additionally, the prospect of making adjustments to family life was opined to deter females from pursuing the APN career track because of their role in their family: they were thought to be more ‘family‐orientated’ and less ‘career‐focused’ than males (P13, Male, APN). Moreover, ‘societal pressure [persisted] for men to be the main breadwinner’ (P4, Female, Intern) and for women to bear the responsibility of childbearing.

##### Subtheme: The inevitable ‘glass escalator’

Because nursing was a female‐dominated profession, males who joined nursing would tend to stand out and be more prominent than females, as shared by the participants:

… they [men] also understand that they are more outstanding as males you know, so for example, just within a ward, there's probably going to be only two or three males … Whatever they do is being magnified, if they have a compliment, wow, everybody knows right? *[*sic*]* (P5, Female, Student)

Against such a background, the participants observed that males appeared to be recognized and appreciated more often than females for any given work of similar quality. Furthermore, the participants added that males' opinions appeared to carry more weight than females' in their work environments. Moreover, being the minority in the nursing workforce caused them to receive unintended advantages over females in career advancement. Such observations led to the participants' view that male nurses were on a ‘glass escalator’ and enjoyed accelerated career progression; thus, they were more likely to be identified as potential candidates for the APN career.

#### 3.2.2 Theme 2: The APN career‐track is unpopular among men

Interestingly, though the odds were seemingly in their favour, male nurses were noted to lack interest in becoming APNs. Several factors might have made the APN career less appealing, especially to males.

##### Subtheme: Gender‐stereotyped attributes influencing career choice

Males were generalized to be decisive, analytical, authoritative, and composed, based on which they were thought to be good candidates for leadership roles. Conversely, females were described as nurturing, detail‐oriented, and emotionally‐driven:

… we have some men who are natural leaders, born to be leaders‐type *[*sic*]* (P7, Female, APN)

[institutional leaders] choose men for very big positions like managerial roles … maybe we are females, we are more emotional, I'm a female myself. I can be emotional but males they can control their emotions very well. *[*sic*]* (P16, Female, Student).

Given such perceived personality traits, the participants opined that males in nursing tended to gravitate towards more managerial and leadership‐related career tracks and away from more clinical and education tracks within their organizations. Conversely, the minority of males who did join the APN workforce were usually motivated by their strong interest in clinical work and opportunities to develop further in clinical knowledge and skills. They also saw the APN career track as an opportunity to exercise clinical leadership.

##### Subtheme: Sacrifices in remuneration and career

As aforementioned, all APN candidates would require nomination and funding from their institutions. Although still technically employed by their institutions during the Master of Nursing programme and internship (which spanned about 3 years), most APN candidates experienced minimal salary increments and would not be considered for any promotions:

… the problem is during our [Master of Nursing] studies, we do not get promoted. Whereas your peers that join [the nursing workforce] the same time as you gets promoted. I think that's a very big deterrent for most nurses, not just males, both males, and females. We do hear a lot of unhappiness about it. *[*sic*]* (P10, Female, APN)

Apart from these possible impacts on remuneration and promotion, uncertain career prospects and academic stringency further deterred nurses from becoming APNs. Nurses were concerned with the lack of clear career pathways for APNs, especially compared with the four more structured, conventional career pathways in nursing (clinical, management, education, and research). They might feel more inclined to choose a more structured pathway for which there were established performance indicators. Moreover, educational requirements for the other tracks were more flexible: in some institutions, it was not mandatory for nurse managers, nurse clinicians, nurse educators, or nurse researchers to be Masters‐holders. In sum, although these considerations would be common to both genders, males appeared to be more affected by this, because of expectations on them to provide for their families:

… for guys, opportunity costs and salary are very important. They must support the family. *[*sic*]* (P5, Female, Student)

##### Subtheme: Gender‐based stigmatization of nursing

The participants shared that, given the widespread perception of nursing as an effeminate job, males in the profession were often described with attributes carrying unpleasant connotations:

“It doesn't have that prestige. You tell your relative ‘I'm a nurse’, they say ‘huh? You're a male, why are you a nurse, are you a sissy?’. Quite sadly, people also do not see nursing as a profession. *[*sic*]* (P12, Male, APN)

Coupled with the public's negative perceptions, such gender‐based stigmatization of nursing led to low morale among male nurses, who were apprehensive about their career longevity in nursing, as shared by the participants. Consequently, they did not feel the need to develop further in their nursing career, such as the pursuit of the APN career track. Some even eventually left nursing for another profession:

… some of the guys went to medical school, and some of them went to do property [real estate agents]. Some of them went to become insurance agents. Most of my [male] friends, they venture into business. *[*sic*]* (P12, Male, APN).

#### 3.2.3 Theme 3: Balancing the gender gap in the APN workforce

The participants did not find males' lack of interest in the APN career track concerning. Nonetheless, they did see value in disrupting the status quo of their low numbers in the APN workforce.

##### Subtheme: Male APNs are ‘good‐to‐have’ and not a ‘must‐have’

The scarcity of males in the APN workforce was not perceived to be unsettling. During the discussion on recruiting males into the APN workforce, one participant commented that having more male APNs ‘is not a must‐have but more of a good‐to‐have’ (P10, Female, APN). Such a view was shared by most other participants because the gender of the APN hardly influenced the scope and outcomes of practice:

I feel it [gender of the APN] doesn't really matter. Because what we [APNs] do, our scope of practice, is the same for both male and female APNs. *[*sic*]* (P11, Male, APN).

Instead, there was a general need to recruit more committed and passionate APNs, both males and females:

… we need more APNs rather than need more male APN. *[*sic*]* (P13, Male, APN)

it really depends on whether they have that, in Chinese we call it, 心和力 [passion and commitment] you know? Like they have that commitment and that passion to take on the role of the APN … doesn't need to be male or female … *[*sic*]* (P7, Female, APN)

Nevertheless, there was consensus among the participants that it would be healthy to have gender diversity within the APN workforce. With males and females perceived to have gender‐specific traits and strengths, gender diversity within the workforce could promote better exchange of ideas:

… regardless of profession, it's always good to have a gender balance because different genders bring different characteristics. *[*sic*]* (P4, Female, Intern)

##### Subtheme: Good role models and better articulation of the role

To address the gender disparity in the APN workforce, the recruitment of males should be improved. Many participants cited the lack of role clarity as a deterrent for males to pursue the APN career track. It followed that exemplary APN role models would inspire early‐career nurses to become APNs themselves:

They'll [early career nurses] be like ‘oh, what a good role model. I look forward to becoming like that!’ … we have a male APN in our institution, everybody looks up to him because of the knowledge that he has to offer … the amount he has to teach. But I think his gender plays a part as well. Having a male APN does impact the number of people who wants to join the APN course. *[*sic*]* (P16, Female, Student]

To improve role clarity of APNs, the participants advocated the need to more accurately publicise and ‘rebrand’ it as a ‘knowledge‐intensive occupation’:

APNs should be advertised in Singapore as nurses who have the knowledge and skills that go beyond making people feel better. APNs have the clinical expertise and it is a knowledge‐intensive occupation. I think this can attract more men into the nursing profession … it's not just about us being caring, it is a knowledge‐intensive occupation. *[*sic*]* (P4, Female, Intern)

##### Subtheme: ‘De‐gendering’ the stigmatization of nursing

Through better gender diversity within the APN workforce, the participants believed that the stigma of nursing being an effeminate job would be reduced and that nursing would be viewed as an inclusive profession with diverse demographics:

… it tells people we are more open to having different types of people perform what used to be a very feminine role within the healthcare system. *[*sic*]* (P10, Female, APN)

With greater inclusivity in the APN workforce, patients, the public, and even healthcare colleagues would notice that males were developing professionally and thriving in nursing. This might contribute to reducing the gender‐based stigmatization of nursing.

If they have more male representation in the APN workforce, when patients visit the hospital, they see men doing a wonderful job in the hospital and are impressed by their care received during their hospital stay. Subsequently, the patient will change their perspectives about nursing. *[*sic*]* (P6, Male, Student)

## 4 DISCUSSION

At present, this qualitative inquiry is the first of its kind in Asia to explore the gender disparity and recruitment of males in the APN workforce from the perspectives of males and females pursuing or in the midst of their APN careers. This study shares important insights into addressing the gender gap in the APN workforce.

Much of the existing literature drew attention to the discrimination men experience, in the form of gender role conflicts and stereotypes, throughout their nursing career (Brody et al., 2017; Younas et al., 2019). Interestingly, in this study, although gender‐based stigmatization of nursing is ascertained as a longstanding issue in Singapore, men in nursing did not seem to experience discrimination with regard to professional development. Instead, males in nursing appear to be beneficiaries of opportunistic advantages in professional development. Despite the objectivity in the selection of APN candidates, males are more likely to be identified as potential candidates for APN training than their female counterparts. This can be attributed to the dominant role of females in childbearing and caregiving (Holahan, 1994) and is true even among highly intelligent females with successful careers. A systematic review (Schlegler, 2022) on the professional state of gifted adults has reported that females would experience significant changes in their careers after the age of 35 years. Females were observed either to transit from being gainfully employed before starting a family to becoming homemakers or to remain gainfully employed without much career aspiration. Moreover, gifted females have also been reported to be more likely than gifted males to face trade‐off decisions where they had to choose between their career or family: ultimately, they would gravitate towards a career that made allowances for their family planning (Schlegler, 2022). Such literature validates the findings in this study, considering that most RNs start their APN preparatory training in their 30s (Woo et al., 2020).

The term ‘glass escalator’ first introduced by Williams (1992), describes the underlying advantage men enjoy in female‐dominated professions that facilitates their career progression. The phenomenon of such a ‘glass escalator’ has been underscored in this study. According to a few earlier studies, men may feel different or out of place among their female nursing colleagues but often do not feel discriminated against (Smith et al., 2020; Zeb et al., 2020). Conversely, as the minority in nursing, males have been reported by the participants to be more prominent than their females, and their positive work performances are more likely to be noticed. Such findings are echoed in Asian (Zhang & Tu, 2020) and Western (Smith et al., 2020) nursing populations. Moreover, across cultures, males in nursing have been observed to receive greater recognition from their managers than females. These observations therefore, lead to their favourable positions for career advancements (Smith et al., 2020; Younas et al., 2022; Zhang & Tu, 2020).

To address the ‘glass escalator’ phenomenon, an obvious implication for the management of staff would be to slow it down strategically (Brandford & Brandford‐Stevenson, 2021). However, this is superfluous in the context of APN recruitment of males in Singapore. Elucidated in this study is males' disinterest in the APN role, as explained by the achievement‐orientated traits of males such as decisiveness, aggressiveness, independence, and firmness (Heilman, 2001), which are found among males in nursing too. This, therefore, causes them to prefer managerial positions, for which the career progression is more certain, in their organizations.

Ironically, despite the apparent opportunistic advantages of males in nursing, the impact of gender‐based stigmatization of nursing remains evident. This longstanding stigmatization makes it challenging for males to adapt to the female‐dominated nursing culture, which compromises their delivery of patient care and ultimately reduces their job satisfaction (Finnegan, 2019; Younas et al., 2022). Such negative implications deter males from staying in the profession long enough to become APNs. Therefore, as demonstrated by our findings, addressing the gender gap within the APN workforce fosters a healthier and more inclusive working environment.

As elucidated in this study, experiences for males in nursing are complex, spanning from the privilege of being on the ‘glass escalator’ to the stigmatization for being in nursing. Although this is so, recruiting more males into the APN workforce may address the general shortfall in the profession. Addressing this shortfall may inevitably improve nurses' working conditions (Clifton et al., 2020).

Lastly, within the context of this study, nurses have expressed apprehension on becoming APNs because of the lack of role clarity (Woo et al., 2019, 2020). Hence, a delineation of the APN role may facilitate the recruitment of APNs. More specifically, to reduce the stigmatization and encourage gender diversity in the workforce, it may be propitious to have positive male role models mentor nurses (Armitage, 2013). This has also been demonstrated in this study. Intentional mentoring of male nurses through a ‘buddy’ system and informal meetings may be good avenues for men to share experiences, which can help reduce feelings of isolation and exclusion. Additionally, nursing leaders and administrators should be more proactive in creating a positive and inclusive environment where male nurses are supported and accepted by patients and peers and improve their visibility (Younas et al., 2022). Such strategies are envisioned to retain males sufficiently long in nursing to pursue the APN career.

### 4.1 Limitations

One limitation of this study was the purposive sampling of the participants, which was distinctive of qualitative inquiry. Though they were in various APN career stages, the participants were all recruited from Singapore, which may limit the generalizability of the findings to other countries. Nonetheless, the aim of this study was not to generalize but to promote the transferability of knowledge from one context to another. Our findings provide pioneering insights that can be transferred to other contexts in similar stages of APN development.

## 5 CONCLUSIONS

Although males in nursing enjoy opportunistic advantages in entering the APN workforce, they are not interested in the role and not staying long enough in the nursing profession to become APNs. This paper offers insights into the poor recruitment of males in the APN workforce and suggestions to combat that. Further inquiry is required to understand how regulatory bodies and nursing institutions may promote a more inclusive environment that reduce gender stigmatization.

### 5.1 Implications for nursing management

Nurse managers should be more cognizant of the different social stereotypes faced by males and females in nursing. With such awareness, they can be more supportive and less biased in career counselling and job appraisals. In light of the need to improve APN recruitment, institutional leaders and policy makers need to be more proactive in recruiting RNs into the APN preparatory training. Greater flexibility in the completion of the training for female candidates with children may be advantageous to increasing interest in the APN career. Positive role modelling and clear articulation of the APN role may also improve the recruitment of both male and female candidates into the APN workforce.

## CONFLICTS OF INTEREST

The authors declare no conflicts of interest.

## ETHICS STATEMENT

All research activities of the study were approved by the Institutional Review Board of the National University of Singapore (NUS‐IRB‐2021‐71).

## Supporting information


**Data S1.** Supporting Information

## Data Availability

The data that support the findings of this study are available on request from the corresponding author. The data are not publicly available due to privacy or ethical restrictions.
